# Benzo(a)pyrene induces similar gene expression changes in testis of DNA repair proficient and deficient mice

**DOI:** 10.1186/1471-2164-11-333

**Published:** 2010-05-26

**Authors:** Nicole Verhofstad, Jeroen LA Pennings, Conny ThM van Oostrom, Jan van Benthem, Frederik J van Schooten, Harry van Steeg, Roger WL Godschalk

**Affiliations:** 1 Department of Health Risk Analysis and Toxicology, School for Nutrition, Toxicology and Metabolism, Maastricht University, PO box 616, 6200 MD Maastricht, the Netherlands; 2 Laboratory for Health Protection Research, National Institute for Public Health and the Environment (RIVM), PO box 1, 3720 BA Bilthoven, the Netherlands

## Abstract

**Background:**

Benzo [a]pyrene (B[a]P) exposure induces DNA adducts at all stages of spermatogenesis and in testis, and removal of these lesions is less efficient in nucleotide excision repair deficient *Xpc*^-/- ^mice than in wild type mice. In this study, we investigated by using microarray technology whether compromised DNA repair in *Xpc*^-/- ^mice may lead to a transcriptional reaction of the testis to cope with increased levels of B[a]P induced DNA damage.

**Results:**

Two-Way ANOVA revealed only 4 genes differentially expressed between wild type and *Xpc*^-/- ^mice, and 984 genes between testes of B[a]P treated and untreated mice irrespective of the mouse genotype. However, the level in which these B[a]P regulated genes are expressed differs between Wt and *Xpc*^-/- ^mice (p = 0.000000141), and were predominantly involved in the regulation of cell cycle, translation, chromatin structure and spermatogenesis, indicating a general stress response. In addition, analysis of cell cycle phase dependent gene expression revealed that expression of genes involved in G1-S and G2-M phase arrest was increased after B[a]P exposure in both genotypes. A slightly higher induction of average gene expression was observed at the G2-M checkpoint in *Xpc*^-/- ^mice, but this did not reach statistical significance (P = 0.086). Other processes that were expected to have changed by exposure, like apoptosis and DNA repair, were not found to be modulated at the level of gene expression.

**Conclusion:**

Gene expression in testis of untreated *Xpc*^-/- ^and wild type mice were very similar, with only 4 genes differentially expressed. Exposure to benzo(a)pyrene affected the expression of genes that are involved in cell cycle regulation in both genotypes, indicating that the presence of unrepaired DNA damage in testis blocks cell proliferation to protect DNA integrity in both DNA repair proficient and deficient animals.

## Background

Exposure to chemicals like benzo(a)pyrene (B[a]P) can lead to structural changes in DNA and as a consequence to the development of diseases with a genetic basis [1]. Changes in the DNA sequence can be induced by exposure to chemicals during life, but may also be inherited via mutations in the spermatogonial stem cells; in that way increasing the risk of developing abnormalities or diseases in the offspring [2,3]. The mutagenic potential of B[a]P in male germ cells, however, has still not been fully established. B[a]P related DNA damage was observed at all stages of spermatogenesis and in testis [4,5], but it is largely unknown how germ cells deal with DNA damage to protect their genetic material, and to prevent the accumulation of mutations in the germ line.

Spermatogenesis is carefully controlled to produce mature spermatozoa from spermatogonial stem cells in three major stages; the mitotic stage, the meiotic stage and the maturation stage. Germ cells are susceptible for the induction of mutations during mitotic and meiotic divisions, because cell turnover is a prerequisite for fixation of DNA damage into mutations. However, it is likely that several processes prevent the occurrence of gene mutations in male germ cells; for example, DNA damage can be removed by DNA repair mechanisms, of which nucleotide excision repair (NER) is considered to be the most relevant repair mechanism for bulky DNA adducts formed by reactive metabolites of B[a]P. Two NER mechanisms have been described: global genome repair (GGR) eliminates bulky DNA lesions in the entire genome, whereas transcription coupled repair (TCR) specifically removes lesions that block RNA synthesis [6]. In a previous study, we observed that GGR/NER plays an important role in the removal of B[a]P induced DNA adducts in the testis, especially in the first week after exposure [5]. B[a]P induced DNA adduct levels in the testis were significantly different between Wt and *Xpc*^-/- ^mice, especially at 4 days after a single exposure to B[a]P (0.69 ± 0.16 and 1.84 ± 0.70 adducts per 10^8 ^nucleotides in Wt and *Xpc*^-/- ^mice, respectively). Therefore, in an attempt to reveal the responses in the testis to this damage, we investigated by using microarrays the changes in gene expression induced by B[a]P in testis from Wt and *Xpc*^-/- ^male mice, 4 days after exposure to B[a]P. Since *Xpc*^-/- ^mice lack one of the most important protective mechanisms against B[a]P induced DNA damage, we expected a different (adaptive) transcriptional response in these mice as compared to their Wt counterparts.

## Results

### Gene expression profiling

**Figure 1:**
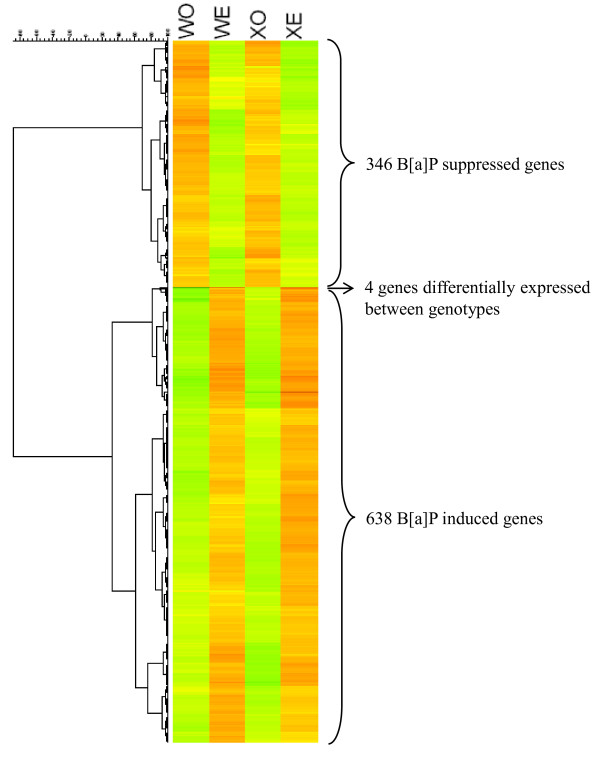
**Cluster analysis of differentially expressed genes**. Differentially expressed genes were clustered based on Euclidian Distance and Ward linkage using GeneMaths. WO, Wt control. WE, Wt exposed. XO, *Xpc*^-/- ^control. XE, *Xpc*^-/- ^exposed.

Two-way ANOVA revealed 984 regulated genes that were differentially expressed between treated and untreated mice (FDR 5%), of which 638 genes were increased and 346 genes were decreased by B[a]P exposure. Only 4 genes (*Xpc*, *Cml2*, *D6Mm5e *and 2610209M04Rik) were differentially expressed between unexposed Wt and unexposed *Xpc*^-/- ^mice (FDR 5%), and they are all located at the same site of chromosome 6. Of these 4 genes, *Xpc*, *Cml2 *and *D6Mm5e *were decreased in expression in *Xpc*^-/- ^mice as compared to the Wt mice, with higher gene expression levels of *Cml2 *and *D6Mm5e *than the very low levels of *Xpc*. On the other hand, 2610209M04Rik was increased in *Xpc*^-/- ^mice. This confirms the absence of a functional *Xpc *gene in the *Xpc*^-/- ^genotype, and shows that closely linked genes have significantly altered expression patterns. Finally, our results show that there is only a modest difference between the gene expression patterns in testis of B[a]P treated Wt and *Xpc*^-/- ^mice. Nonetheless, a paired T-test showed that the level in which these B[a]P regulated genes are expressed differs between Wt and *Xpc*^-/- ^mice (p = 0.000000141). The clustering of the combined hits is presented in Figure 1.

### Analysis of pathway regulation

The list of the 638 genes that were induced by B[a]P suggested, by using DAVID/EASE, a significant enrichment for several Gene Ontology (GO) biological processes and Swiss-Prot_Protein Information Resource (SP_PIR) terms, such as 'cell division' (p = 0.000000082), 'mitosis' (p = 0.00000037), 'chromosome segregation' (p = 0.000085) and 'spermatogenesis' (p = 0.00024) (Additional file 1). The 346 genes that were down regulated by B[a]P exposure also showed an enrichment for several pathways, including 'oxidative phosphorylation' (p = 0.0011) (Additional file 2). In addition, two GO terms for the cellular components mitochondrial part (p = 0.001) and mitochondrion (p = 0.0011), were enriched among B[a]P suppressed genes, which is in line with the inhibition of the oxidative phosphorylation process.

The combined list of B[a]P (both up and down) regulated genes showed an enrichment of 110 GO and SP_PIR terms (Additional file 3), including cell cycle, translation, chromatin structure and spermatogenesis, which we already found to be enriched among the up regulated genes only. Therefore, we used these 4 processes to further select genes and to subsequently compare the two genotypes regarding their gene expression ratios between B[a]P treated *vs*. control groups. An overview of the gene expression ratio distribution is given in Table 1. 46 differentially regulated genes were involved in the cell cycle (Table 2), of which 20 genes were induced to a higher extent in *Xpc*^-/- ^mice, whereas only 12 of these genes were expressed to a higher extent in Wt mice. Regarding the genes that were inhibited in their expression, 6 genes were more suppressed in *Xpc*^-/- ^mice, and 8 genes in Wt mice as compared to animals of the other genotype. In addition, we found 37 genes that are involved in the chromatin structure (Additional file 4), of which 20 genes were more induced in *Xpc*^-/- ^mice, and 8 genes in Wt mice. Only 8 chromatin structure related genes were more suppressed in Wt mice, as compared to 1 gene in *Xpc*^-/- ^mice. Among the spermatogenesis related genes (Additional file 5), we only found differences between Wt and *Xpc*^-/- ^animals among the B[a]P suppressed genes; 9 genes were more suppressed in Wt mice compared to 1 gene in *Xpc*^-/- ^mice as compared to animals of the other genotype. Taken together, these results indicate that gene expression differences between Wt and *Xpc*^-/- ^mice are small, and that the induction of genes involved in cell division regulating processes was stronger in *Xpc*^-/- ^than in Wt mice. We did not find relevant differences in average gene expression ratios between *Xpc*^-/- ^and Wt mice among translation related genes, that are given in Additional file 6.

**Table 1:** Comparison of gene expression ratios.

Pathway	Total # genes	Uw	Ux	Dw	Dx
Cell cycle	46	12	20	8	6
Chromatin structure	37	8	20	8	1
Translation	59	18	22	7	12
Spermatogenesis	32	10	12	9	1

Average gene expression ratios for genes involved in significantly regulated pathways are compared between Wt and *Xpc*^-/- ^mice. The table shows the number of genes that are up or down regulated to a higher extent after B[a]P exposure in either genotype.

Uw, up regulated to a higher extent in Wt mice. Ux, up regulated to a higher extent in *Xpc*^-/- ^mice. Dw, down regulated to a greater extent in Wt mice. Dx, down regulated to a greater extent in *Xpc*^-/- ^mice.

**Table 2:** Differentially regulated genes regarding the cell cycle process after B[a]P exposure.

Symbol	Gene Name	Wt	*Xpc*^-/-^
Bin3	bridging integrator 3	1.13	1.31
Brca2	breast cancer 2	-1.35	-1.34
Brms1	breast cancer metastasis-suppressor 1	-1.18	-1.34
Calr	calreticulin	-1.26	-1.31
Ccnb2	cyclin B2	-1.24	-1.16
Ccng2	cyclin G2	1.23	1.32
Ccnk	cyclin K	1.21	1.23
Cdc25c	cell division cycle 25 homolog C (S. cerevisiae)	-1.09	-1.30
Cdc27	cell division cycle 27 homolog (S. cerevisiae)	1.41	1.31
Cdc42	cell division cycle 42 homolog (S. cerevisiae)	1.34	1.50
Cdca1	cell division cycle associated 1	1.24	1.50
Cdca5	cell division cycle associated 5	1.18	1.30
Cdca8	cell division cycle associated 8	-1.24	-1.11
Cdk5rap3	CDK5 regulatory subunit associated protein 3	-1.24	-1.23
Cetn1	centrin 1	1.46	1.31
Cit	citron	1.20	1.35
Cks2	CDC28 protein kinase regulatory subunit 2	1.29	1.36
Cul3	cullin 3	1.28	1.18
Dhcr24	24-dehydrocholesterol reductase	1.50	1.43
Dnajc2	DnaJ (Hsp40) homolog, subfamily C, member 2	-1.42	-1.23
Fzr1	fizzy/cell division cycle 20 related 1 (Drosophila)	1.44	1.39
Gspt1	G1 to S phase transition 1	1.37	1.31
Hells	helicase, lymphoid specific	1.43	1.46
Incenp	inner centromere protein	1.17	1.29
Kif20a	kinesin family member 20A	1.54	1.41
Macf1	microtubule-actin crosslinking factor 1	1.16	1.49
Mad2l2	MAD2 mitotic arrest deficient-like 2 (yeast)	1.19	1.27
Mphosph6	M phase phosphoprotein 6	1.30	1.26
Mtus1	mitochondrial tumor suppressor 1	-1.22	-1.19
Nasp	nuclear autoantigenic sperm protein (histone-binding)	1.34	1.38
Nek4	NIMA (never in mitosis gene a)-related expressed kinase 4	1.31	1.37
Pin1	protein (peptidyl-prolyl cis/trans isomerase) NIMA-interacting 1	1.31	1.26
Ppp3ca	protein phosphatase 3, catalytic subunit, alpha isoform	-1.19	-1.22
Pten	phosphatase and tensin homolog	1.32	1.31
Rad17	RAD17 homolog (S. pombe)	1.22	1.27
Ranbp1	RAN binding protein 1	-1.24	-1.23
Rbm5	RNA binding motif protein 5	1.34	1.39
Sgol1	shugoshin-like 1 (S. pombe)	1.26	1.20
Smarcb1	SWI/SNF related, matrix associated, actin dependent regulator of chromatin, subfamily b, member 1	1.27	1.33
Sycp3	synaptonemal complex protein 3	1.34	1.30
Tacc3	transforming, acidic coiled-coil containing protein 3	1.31	1.34
Tipin	timeless interacting protein	-1.22	-1.24
Trrap	transformation/transcription domain-associated protein	-1.26	-1.22
Tubb6	tubulin, beta 6	-1.33	-1.44
Ube2i	ubiquitin-conjugating enzyme E2I	1.17	1.30
Zw10	ZW10 homolog (Drosophila), centromere/kinetochore protein	1.23	1.30

46 differentially regulated genes regarding the cell cycle process. Results are presented as average gene expression ratios between exposed and control samples.

Among the 110 GO and SP_PIR terms that were obtained from the combined list of B[a]P (both up and down) regulated genes, we again found a few mitochondria related terms, such as the cellular components 'mitochondrial part', 'mitochondrial envelope' and 'mitochondrial membrane', and the SP_PIR Keyword 'mitochondrion'. Furthermore, we found the SP_PIR keyword 'chaperone', which involves genes that are known to be activated during a stress response.

### Cell cycle phase activity

**Figure 2:**
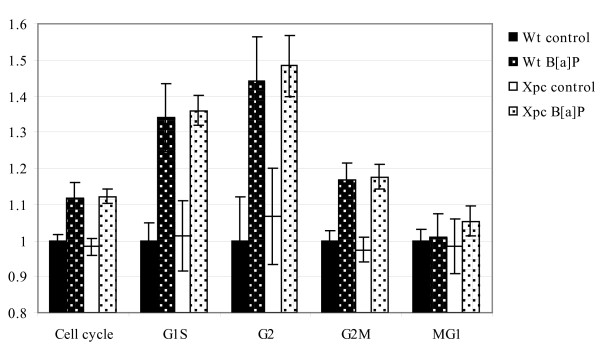
**Average gene expression levels of cell cycle related gene sets**. The setwise average expression levels of genes involved in cell cycle regulation and regulation during specific phases of the cell cycle. Error bars presented are based on the expression levels of individual mice. The average expression level of Wt control mice is set at 1.

The setwise average gene expression levels of cell cycle related gene sets were calculated for sets of genes related to specific cell cycle phases, in order to study cell cycle phase activity (Figure 2). The sets used were based on GO functional annotation for the complete cell cycle or genes found specifically expressed in a particular cell cycle phase, reported by both Bar-Joseph *et al*. [7] and Whitfield *et al*. [8]. Average gene expression levels of all cell cycle genes were lower in *Xpc*^-/- ^mice than in Wt mice (p = 0.024) in the absence of exposure to B[a]P. However, after B[a]P exposure gene expression levels of the same cell cycle related genes were increased to comparable levels in both genotypes. When focusing on specific cell cycle phases, we observed that the average gene expression levels of genes within the cell cycle transition between G1 and S was similar in Wt and *Xpc*^-/- ^control mice, and again increased after B[a]P exposure to equal levels (p = 0.937). On the other hand, the average level of expression of genes involved in the G2-M phase transition was initially lower in *Xpc*^-/- ^control mice compared to the Wt control mice, and gene expression levels were increased by B[a]P exposure to a higher extent in *Xpc*^-/- ^mice compared to the Wt mice. Table 3 shows the gene expression ratios of the 18 genes involved in the G2-M phase transition, indicating that 11 out of these 18 genes were more induced in *Xpc*^-/- ^mice after B[a]P exposure as compared to Wt mice (p = 0.086). Although these differences between Wt and *Xpc*^-/- ^mice are not significant, they are consistent since the majority of genes respond into the same direction, and represent an observed trend towards a more pronounced response to B[a]P in *Xpc*^-/- ^mice. Within the M-G1 phase, we observed a low impact of B[a]P exposure in both genotypes, as compared to the other cell cycle phases. Also within this cell cycle phase, the response to B[a]P was more pronounced in *Xpc*^-/- ^mice than in Wt mice, although this was based on the expression of 4 genes only (p = 0.300).

**Table 3:** Differentially regulated genes involved in G2-M phase transition.

Symbol	Gene Name	Wt	*Xpc*^-/-^
Arl4*	ADP-ribosylation factor-like 4	1.15	1.20
Asf1b*	ASF1 anti-silencing function 1 homolog B	1.24	1.25
Ccnb2	cyclin B2	-1.24	-1.16
Cdc25c	cell division cycle 25 homolog C (S. cerevisiae)	-1.09	-1.30
Cdc42ep4	CDC42 effector protein (Rho GTPase binding) 4	1.33	1.29
Cdca8	cell division cycle associated 8	-1.24	-1.11
Cit*	citron	1.20	1.35
Cks2*	CDC28 protein kinase regulatory subunit 2	1.29	1.36
Ect2*	ect2 oncogene	1.25	1.45
Got1	glutamate oxaloacetate transaminase 1, soluble	1.24	1.24
Gpsm2*	G-protein signalling modulator 2 (AGS3-like)	1.23	1.33
Kpna2	karyopherin (importin) alpha 2	1.35	1.30
Map3k6*	mitogen-activated protein kinase kinase kinase 6	1.25	1.35
Psip1*	PC4 and SFRS1 interacting protein 1	1.17	1.30
Rrm1*	ribonucleotide reductase M1	1.18	1.22
Tacc3*	transforming, acidic coiled-coil containing protein 3	1.31	1.34
Tdp1	tyrosyl-DNA phosphodiesterase 1	1.28	1.21
Tubd1*	tubulin, delta 1	1.26	1.30

Gene expression ratios are presented of 18 differentially regulated genes involved in G2 to M phase transition based on microarray results. These 18 genes, of which 3 were down regulated and 15 were up regulated after B[a]P exposure, showed a trend towards a more pronounced induction in *Xpc*^-/- ^mice as compared to Wt mice (p = 0.086). However, a less extensive down regulation is not the same as a greater induction. Therefore, an additional p-value was calculated for the 15 up regulated genes, showing that 11 (indicated with *) out of these 15 up regulated genes are more induced in *Xpc*^-/- ^than in Wt mice (p = 0.024).

### Validation with quantitative Real-Time PCR

Quantitative Real-Time PCR (qPCR) was performed on 4 selected genes that are involved in the G2-M phase transition to validate the gene expression changes in Wt *vs*. *Xpc*^-/- ^mice after B[a]P exposure. Three genes (*Cit*, *Ect2 *and *Psip1*) were selected because their average gene expression ratios were most different between Wt and *Xpc*^-/- ^mice based on the microarray results, and *Asf1b *was selected as a control because of the small difference between Wt and *Xpc*^-/- ^mice. After gene expression changes were normalised to the *Hprt1 *reference, qPCR data of the 3 strongly differentially expressed genes indeed showed a higher induction of gene expression after B[a]P exposure in *Xpc*^-/- ^mice (ratios are 1.15 *vs*. 1.18, 1.00 *vs*. 1.05 and 1.00 *vs*. 1.04 for *Cit*, *Ect2 *and *Psip1*, in Wt and *Xpc*^-/- ^mice, respectively). However, the effect of B[a]P exposure was smaller on basis of the qPCR expression data. QPCR data of *Asf1b *showed a down regulation after B[a]P exposure in both Wt and *Xpc*^-/- ^mice (ratios are 0.94 and 0.98, respectively), and did therefore, not confirm the microarray results. Overall, 3 out of 4 selected genes confirmed the microarray results found within the G2-M phase, showing that B[a]P induces marginally higher gene expression changes in *Xpc*^-/- ^than in Wt mice.

## Discussion

B[a]P exposure leads to the formation of DNA adducts in testis and sperm of exposed male mice and humans [4,5]. These pro-mutagenic DNA adducts are usually removed by DNA repair mechanisms to prevent the accumulation of mutations. In a previous study, we found that there is a difference in DNA adduct removal between Wtand *Xpc*^-/- ^mouse testes [5]. In the first week after exposure to B[a]P, we observed more DNA adducts in testis of *Xpc*^-/- ^mice, and a slower decline of DNA adducts over time. In addition, we showed that spermatogonial stem cells rely on nucleotide excision repair for the complete removal of DNA adducts during spermatogenesis [5]. This suggests that GGR/NER plays an important role in mice in protecting the testis and sperm cells from DNA damage by B[a]P. In the present study, we used a microarray approach to study whether these differences in DNA adduct removal between Wt and *Xpc*^-/- ^mice are accompanied by differences in gene expression, thereby elucidating adaptive responses towards the induction of heritable DNA damage in testis. We chose to analyze gene expression after 4 days, since at that time point DNA adduct levels differed more between Wt and *Xpc*^-/- ^mice compared to Day 1, and still differed at Day 7 [5].

Two-Way ANOVA indicated that there are only minor expression differences between *Xpc*^-/- ^and Wt mice. No more than 4 genes (*Xpc*, *Cml2*, *D6Mm5e *and 2610209M04Rik) located in a 10 MB locus on chromosome 6, were differentially expressed between the two genotypes. Although the expression levels of *Cml2*, *D6Mm5e *and 2610209M04Rik were higher as compared to the very low levels of *Xpc*, the fact that these genes are located in proximity to the *Xpc *gene on chromosome 6 suggests that mutating the *Xpc *gene has changed the genomic or regulatory structure at that particular site and therefore the expression of these 3 genes. Another explanation could be that these 3 genes are of an Ola129 genetic background, which is used to create the *Xpc *mutant, and are for some unknown reason differently expressed [9].

The main differences were found between the B[a]P exposed and control mice, regardless of genotype. 984 Genes were differentially regulated after B[a]P exposure and these were enriched for genes involved in cell cycle, translation (ribosomes), chromatin structure and spermatogenesis. Besides these pathways, other more general processes were found to be affected (Additional file 3). Regulated processes regarding mitochondrial function (energy metabolism), chaperones, and the aforementioned changes in cell cycle seem to indicate a general stress response after exposure to B[a]P in testes of both genotypes. In addition, the altered expression of mitochondria related genes is indicative for changes in the oxidative phosphorylation; this process was also found to be affected among the B[a]P down regulated genes, and the genes for several ATP synthase subunits, NADH dehydrogenase (ubiquinone) subunits, cytochrome c and cytochrome c oxidase were differentially regulated (Additional file 7). Therefore, the motility and the fertilizing capacity of the sperm cells might have been affected, since mitochondria in sperm are responsible for the energy supply required to propel the flagellum [10]. A change in mitochondrial activity, which leads to less potent sperm cells, may therefore be part of the protective mechanisms against DNA damage in the testis and sperm cells. Indeed, litter sizes of female mice that were fertilized by B[a]P exposed *Xpc*^-/- ^male mice were found to be smaller (data not shown).

B[a]P is a well known mutagen and carcinogen, and mostly studied for its capacity to form bulky DNA adducts, which are mainly repaired by NER. By changing the NER capacity in *Xpc*^-/- ^mice, we expected to find additional mechanisms affected by B[a]P exposure that could possibly protect the testis and male germ cells against the formation of heritable DNA damage, as a back up for the lack of GGR/NER. However, pathways involving DNA repair (other than NER) or apoptosis were not significantly enriched among our regulated genes. Nonetheless, we did find 16 differentially regulated genes that are involved in DNA repair (GO:0006281~DNA repair) (Table 4), and at least 9 out of these 16 genes are related to repair of double strand breaks and/or homologous recombination. This suggests that exposure to B[a]P may cause double strand breaks, for instance by the collapse of the replication fork in replicating cells. Indeed, the number of γH2AX foci, which is a hallmark for double strand break formation, was reported to be increased by exposure to B[a]P [11]. Although the pathway involving apoptosis was not significantly enriched among our regulated genes, we found 32 apoptosis-related genes to be differentially expressed after B[a]P exposure (Table 5). In testes, apoptosis is a common process to discard excessive germ cells, or to remove severely damaged germ cells [12]. From our data it is hard to conclude whether apoptosis is elicited by B[a]P exposure, since we found only a small number of differentially expressed genes after B[a]P exposure and both pro-apoptotic and anti-apoptotic signals were found. For example, cytochrome c (*Cycs*) was up regulated after exposure in both Wt and *Xpc*^-/- ^mice, which could be indicative for the initiation of the intrinsic apoptotic pathway. On the other hand, the expression of the anti-apoptotic gene *Faim *(Fas apoptotic inhibitory molecule) was also up regulated after B[a]P exposure, indicating an inhibitory effect on the apoptotic pathway [13]. Several aspects should be considered for the interpretation of transcriptional data regarding the regulation of apoptosis after B[a]P exposure; 1) the large number of apoptotic cells in normal testis will make it difficult to measure subtle changes in the apoptotic response after a single B[a]P exposure, 2) the apoptotic response after B[a]P exposure is an acute response that is probably hard to assess 4 days after exposure, and 3) the activation of the different caspases leading to fragmentation of DNA occurs by cleavage of procaspases already stored in the cell, a process that is unlikely to be found at the transcriptional level [14]. It should be noted that this last point may also hold true for other processes such as DNA repair, which also relies on activation and transport of already existing proteins.

**Table 4:** Differentially regulated genes concerning DNA repair.

Symbol	Gene Name	Wt	*Xpc*^-/-^
5830483C08Rik	riken cDNA 5830483c08 gene	1.13	1.17
Als4	amyotrophic lateral sclerosis 4 homolog (human)	1.28	1.44
Asf1a	asf1 anti-silencing function 1 homolog a (s. cerevisiae)	-1.34	-1.24
Brca2*	breast cancer 2	-1.35	-1.34
Cry1	cryptochrome 1 (photolyase-like)	1.19	1.29
Cspg6*	chondroitin sulfate proteoglycan 6	1.39	1.40
Fbxo18	f-box protein 18	1.13	1.26
Hmgn1	high mobility group nucleosomal binding domain 1	1.36	1.36
Mpg*	n-methylpurine-dna glycosylase	1.38	1.36
Polg	polymerase (dna directed), gamma	1.36	1.42
Rad17*	rad17 homolog (s. pombe)	1.22	1.27
Rad52*	rad52 homolog (s. cerevisiae)	-1.21	-1.16
Ssrp1*	structure specific recognition protein 1	1.13	1.30
Tdp1*	tyrosyl-dna phosphodiesterase 1	1.28	1.21
Xpc	xeroderma pigmentosum, complementation group C	-1.07	-1.11
Xrcc2*	x-ray repair complementing defective repair in chinese hamster cells 2	-1.28	-1.16
Xrcc5*	x-ray repair complementing defective repair in chinese hamster cells 5	1.23	1.35

16 differentially regulated genes concerning DNA repair. Results are presented as average gene expression ratios between exposed and control samples. *genes associated with double strand breaks and/or homologous recombination

**Table 5:** Differentially regulated genes concerning apoptosis.

Symbol	Gene Name	Wt	*Xpc*^-/-^
1700003F12Rik	riken cdna 1700003f12 gene	1.44	1.45
Atp7a	atpase, cu++ transporting, alpha polypeptide	1.15	1.29
Bag1	bcl2-associated athanogene 1	1.49	1.42
Bag4	bcl2-associated athanogene 4	-1.26	-1.25
Brca2	breast cancer 2	-1.35	-1.34
Cycs	cytochrome c, somatic	1.21	1.37
Dap	death-associated protein	1.31	1.25
Dhcr24	24-dehydrocholesterol reductase	1.50	1.43
Dpf2	d4, zinc and double phd fingers family 2	1.31	1.17
Faim	fas apoptotic inhibitory molecule	1.21	1.20
Hells	helicase, lymphoid specific	1.43	1.46
Hras1	harvey rat sarcoma virus oncogene 1	1.20	1.15
Klrb1c	killer cell lectin-like receptor subfamily b member 1c	-1.09	-1.18
Opa1	optic atrophy 1 homolog (human)	-1.34	-1.17
P2rx1	purinergic receptor p2x, ligand-gated ion channel, 1	1.10	1.17
Pdcd5	programmed cell death 5	-1.22	-1.23
Pim1	proviral integration site 1	-1.17	-1.17
Proc	protein c	1.26	1.22
Pten	phosphatase and tensin homolog	1.32	1.31
Purb	purine rich element binding protein b	1.16	1.30
Qars	glutaminyl-trna synthetase	1.20	1.35
Rragc	ras-related gtp binding c	1.29	1.25
Rybp	ring1 and yy1 binding protein	1.18	1.37
Sh3 kbp1	sh3-domain kinase binding protein 1	-1.37	-1.39
Smndc1	survival motor neuron domain containing 1	1.21	1.35
Sra1	steroid receptor rna activator 1	1.26	1.23
Sycp3	synaptonemal complex protein 3	1.34	1.30
Tax1 bp1	tax1 (human t-cell leukemia virus type i) binding protein 1	1.25	1.08
Tnfrsf4	tumor necrosis factor receptor superfamily, member 4	1.30	1.11
Uaca	uveal autoantigen with coiled-coil domains and ankyrin repeats	1.24	1.29
Uaca	uveal autoantigen with coiled-coil domains and ankyrin repeats	1.14	1.21
Zdhhc16	zinc finger, dhhc domain containing 16	1.25	1.36

32 differentially regulated genes concerning apoptosis. Results are presented as average gene expression ratios between exposed and control samples.

To have a better understanding of the changes in cell cycle regulation, we compared setwise average expression levels of genes related to specific cell cycle phases between the 4 experimental groups. Genes involved in the G1 to S phase transition and the G2 to M phase transition were induced in animals of both genotypes after B[a]P exposure, suggesting that cells may accumulate in both G1 and G2. Cell cycle arrest after B[a]P exposure was previously found, using MCF-7 (human breast carcinoma) and HepG2 (human hepatocarcinoma) cells, and identified altered expression of genes that affect cell cycle regulation [15]. Moreover, cell cycle phases assessed by FACS analysis indeed showed an accumulation of cells in the G1 and G2 phase after B[a]P exposure [15]. It has also been shown that benzo [a]pyrene-7,8-dihydrodiol (BPD) and benzo(a)pyrene-diol-epoxide (BPDE), both metabolites of B[a]P, can induce a G2/M accumulation [16]. All these data are in line with our findings in testis of B[a]P exposed male mice.

The B[a]P induced G1-S arrest did not differ between Wt and *Xpc*^-/- ^mice (p = 0.937). However, expression of G2-M phase genes showed a trend towards a higher induction of these genes in testes of *Xpc*^-/- ^mice after B[a]P exposure (p = 0.086). Therefore, the expression ratios of 4 G2-M phase genes were validated by qPCR, and results of 3 out of these 4 genes confirmed that B[a]P induces slightly higher gene expression changes in *Xpc*^-/- ^mice than in Wt mice. However, the level of induction by B[a]P exposure was higher on basis of microarray analysis, than by qPCR analysis. QPCR could not confirm the induction of *Asf1b *gene expression by B[a]P as shown by microarray analysis, although the difference in gene expression ratios between Wt and *Xpc*^-/- ^mice remained small. Such a small difference in reaction may be explained by the fact that *Xpc*^-/- ^mice are less capable of removing B[a]P induced damage in testis [5], and therefore more damaged cells are arrested (instead of a stronger reaction in each individual cell). This was also observed in a study using UVB irradiated Xpc-deficient epidermal cells [17]; Xpc-deficient cells were blocked in the late S phase due to incomplete DNA replication at sites of damaged DNA [17]. As a result, the regulation of the G2 phase genes might differ slightly between *Xpc*^-/- ^and Wt mice, as found in this study.

Unrepaired DNA damage blocks the cell cycle. However, specialized Y-family DNA polymerases are capable of extending replication beyond lesions in an error-free or error-prone way [17]. For example the expression of DNA polymerase κ (*Polκ*) is under the control of the B[a]P activated arylhydrocarbon receptor (AhR), and more highly expressed in testes than in other tissues [18,19]. It bypasses BPDE-N_2_-dG in an efficient and mostly error-free way [19]. If we assume that DNA repair deficient *Xpc*^-/- ^cells that are present in testes (such as Sertoli cells, Leydig cells and developing sperm cells), in contrast to DNA repair proficient Wt cells, are blocked in the late S phase due to unrepaired damage, then this damage could be passed in an error-free way with progression of the cell cycle and spermatogenesis, but without the actual removal of the blocking lesion. Although *Polκ *was not present among the differentially expressed genes by using Two-Way ANOVA, we found, however, that the expression of *Polκ *was significantly higher in *Xpc*^-/- ^than in Wt mice after B[a]P exposure as assessed by a simple T-test (p = 0.038). However, this differential expression between Wt and *Xpc*^-/- ^mice after B[a]P exposure was found only after correction for the slight initial expression differences found in their unexposed counterparts. Progression of the cell cycle and spermatogenesis, without the actual removal of the DNA lesion is in line with the differences in DNA damage kinetics between *Xpc*^-/- ^and Wt mice found in our previous study [5]. Therefore, we suggest that *Xpc*^-/- ^mice are not per se more sensitive to mutation induction in the testis than Wt mice after B[a]P exposure, and thereby could have equal risks for heritable gene mutations. However, future studies analyzing mutation induction should elucidate the mutagenic potency of B[a]P in this reproductive organ.

## Conclusion

From this study, we can conclude that gene expression in testis of untreated *Xpc*^-/- ^and Wt mice are similar, and this is also the case after exposure to B[a]P. B[a]P exposure, which formed a substantial amount of DNA damage, differentially regulated 984 genes that were enriched for cell cycle, translation, chromatin structure and spermatogenesis. Overall, B[a]P-induced gene expression changes in *Xpc*^-/- ^mice differed from their exposed wild type counterparts (p = 0.000000141), which was also found for stage specific cell cycle gene sets. These gene expression changes in testis may represent a cell cycle block due to the presence of unrepaired damage. Specialized DNA polymerases that are highly expressed in testis may still bypass B[a]P induced DNA damage in an error-free way, and cell cycle/spermatogenesis can thus progress in the presence of DNA lesions.

## Methods

### Animal experiments

Male wild type (C57BL/6) and *Xpc*^-/- ^mice (C57BL/6), as previously described [9,20], were exposed to a single dose of B[a]P (13 mg/kg bw) by oral gavage, and were sacrificed 4 days after exposure. All groups consisted of 5 mice. The dose of 13 mg/kg bw has been used in previous studies and is appropriate to induce detectable DNA adduct levels [21]. The control groups received the solvent sunflower oil and were also sacrificed at day 4 after exposure. Testes were snap frozen in liquid nitrogen and stored at -80°C until analysis. Mice were bred and housed under specific pathogen-free conditions in a 12-hour light-dark cycle at the animal facilities of the Netherlands Vaccine Institute (NVI, Bilthoven, the Netherlands), and received food and water *ad libitum*. Experiments were approved by the NVI's Animal Ethics Committee and were carried out according to their guidelines.

### RNA isolation and purification

After testes had been pulverized using mortar and tamper cooled in liquid nitrogen, TRIzol^® ^Reagent (Invitrogen, Breda, the Netherlands) was added to the material and RNA was isolated using an RNeasy Mini Kit (Qiagen, Venlo, the Netherlands) with DNase treatment according to the manufacturer's protocol with minor modifications. RNA quantity was determined spectrophotometrically and RNA purity was assessed on an Agilent 2100 Bioanalyzer (Agilent Technologies, Amstelveen, the Netherlands). All samples were pure and free of RNA degradation.

### RNA labelling and hybridization on microarrays

Cyanine labelled cRNA was generated by using the Two-Color Microarray-Based Gene Expression Analysis kit from Agilent Technologies according to the manufacturer's protocol. All samples were labelled with Cy5, and 3 samples of unexposed Wt mice were labelled with Cy3, pooled and used as a reference sample.

On the basis of dye incorporation rates, appropriate amounts of Cy5 and Cy3 labelled samples (10 pmol each) were simultaneously hybridized on Agilent 22K Mouse microarrays (Agilent Technologies). After hybridization, slides were washed and dried with N_2 _gas before scanning.

### Image analysis

Slides were scanned on a GenePix^® ^4000B Microarray Scanner (Molecular Devices, Sunnyvale, CA, USA). Cy5 and Cy3 were excited at wavelengths of 635 and 532 nm, respectively. Laser power was set to 100%. The photo multiplier was set to a saturation tolerance of 0.02% to minimize background and saturated spots. The images obtained (resolution 5 micron, 16 bit tiff images) were processed with Imagene 8.0.1 software (Biodiscovery, El Segundo, CA, USA) to measure mean signal intensities for spots and local backgrounds.

### Quality control

Quality control was performed on raw data by means of a scatter plot and MA-plot as well as a normal probability plot to assess signal distribution. Positive (landmark) and negative (blank) spots were used for quality control but not included in further analyses. Microarray spot signal data were normalized in R using a four-step approach of (1) natural log-transformation, (2) quantile normalization of all scans, (3) correction of the sample spot signal for the corresponding reference spot signal and (4) averaging data from replicate spots. Normalized data were visualized by Principal Component Analysis (PCA) for additional quality control. The gene expression data discussed in this publication have been deposited in NCBI's Gene Expression Omnibus [22] and are accessible through GEO Series accession number GSE17979 http://www.ncbi.nlm.nih.gov/geo/query/acc.cgi?acc=GSE17979.

### Data analysis

Significance of differences in gene expression between genotype and/or treatment groups was calculated by a Two-Way ANOVA, where genes with a false discovery rate (FDR) below 5% were selected as differentially expressed. Differentially expressed genes were clustered (based on Euclidian Distance and Ward linkage) using GeneMaths (Applied Maths, St-Martens-Latem, Belgium). By using the DAVID/EASE http://david.abcc.ncifcrf.gov web application, functional annotation and Gene Ontology (GO) term enrichment were determined for three different gene sets, namely B[a]P induced genes, B[a]P suppressed genes and all regulated genes together (FDR < 0.1) [23,24].

Pathway enrichment among B[a]P regulated genes was further used to identify regulated genes within a pathway, and to compare the average gene expression ratios of B[a]P treated *vs*. control groups between the two genotypes. Pathways with related terms for cell cycle, translation, chromatin structure and spermatogenesis, which were already found to be enriched among the up regulated genes, were combined to form 4 groups of genes that were used for this comparison.

For visualization of cell cycle phase activity, the average gene expression for suitable gene sets was calculated. Sets used were based on either GO functional annotation or on genes found specifically expressed in a particular cell cycle phase by both Bar-Joseph *et al*. [7] and Whitfield *et al*. [8], but only used for calculation if they contained at least 4 genes. Setwise average gene expression levels for each experimental group were expressed relative to the Wt control mice, for which the level was set at 1.

### Quantitative Real-Time PCR

For qPCR of 4 selected genes (*ASf1b*, *Cit*, *Ect2 *and *Psip1*) and reference gene *Hprt1*, cDNA was synthesized using the iScript cDNA Synthesis kit (Bio-Rad, Veenendaal, the Netherlands) according to the manufacturer's protocol, starting with 0.5 μg of RNA. QPCR was performed on a MyiQ™ Single-Color Real-Time PCR detection system (Biorad) using the iQ™ SYBR^® ^Green Supermix (Bio-Rad) according to the manufacturer's protocol with minor modifications, 5 μl of 10 times diluted cDNA and 0.3 μM primers in a total volume of 25 μl. Samples were amplified under the following conditions: 95°C for 3 minutes, followed by 40 cycles at 95°C for 15 seconds and 60°C for 45 seconds. PCR was checked for a-specific products by performing a melting curve analysis (65°C-95°C). Data were analyzed using the MyiQ™ Software system (Bio-Rad) and were expressed as average gene expression ratios as compared to controls.

## Authors' contributions

NV carried out the RNA isolation, the labelling and hybridization of the samples and the image analyses, and wrote the manuscript. JP carried out the data and statistical analyses, and has been involved in writing the manuscript. CO carried out the animal experiments. JB, FS and HS participated in the design of the study, interpretation of the data and revision of the manuscript. RG conceived the design of the study, participated in the interpretation of the data, and helped to draft the manuscript. All authors read and approved the final manuscript.

## Supplementary Material

Additional file 1 **Processes enriched among B[a]P induced genes**. The data presented are terms enriched among the B[a]P induced gene list by DAVID/EASE with an FDR < 0.1. Click here for file

Additional file 2 **Processes enriched among B[a]P suppressed genes**. The data presented are terms enriched among the B[a]P suppressed gene list by DAVID/EASE with an FDR < 0.1. Click here for file

Additional file 3 **Processes affected by B[a]P exposure in mouse testis**. The data presented are terms enriched among the B[a]P modulated gene list by DAVID/EASE with an FDR < 0.1. Click here for file

Additional file 4 **Differentially regulated genes after B[a]P exposure involved in Chromatin structure**. 37 differentially regulated genes involved in Chromatin structure. Results are presented as average gene expression ratios between exposed and control samples. Click here for file

Additional file 5 **Differentially regulated genes after B[a]P exposure regulating Spermatogenesis**. 32 differentially regulated genes involved in Spermatogenesis. Results are presented as average gene expression ratios between exposed and control samples. Click here for file

Additional file 6 **Differentially regulated genes after B[a]P exposure involved in Translation**. 59 differentially regulated genes involved in Translation. Results are presented as average gene expression ratios between exposed and control samples. Click here for file

Additional file 7 **Differentially regulated genes after B[a]P exposure involved in Mitochondrial function**. 51 differentially regulated genes involved in mitochondrial function. Results are presented as average gene expression ratios between exposed and control samples. *genes involved in oxidative phosphorylation. Click here for file

## References

[B1] Denissenko MF Pao A Tang M Pfeifer GP Preferential formation of benzo[a]pyrene adducts at lung cancer mutational hotspots in P53 Science 1996 274 430 432 10.1126/science.274.5286.430 8832894

[B2] Somers CM Yauk CL White PA Parfett CL Quinn JS Air pollution induces heritable DNA mutations Proc Natl Acad Sci USA 2002 99 15904 15907 12473746 10.1073/pnas.252499499 PMC138537

[B3] Vilarino-Guell C Smith AG Dubrova YE Germline mutation induction at mouse repeat DNA loci by chemical mutagens Mutat Res 2003 526 63 73 12714184 10.1016/s0027-5107(03)00016-2

[B4] Zenzes MT Bielecki R Reed TE Detection of benzo(a)pyrene diol epoxide-DNA adducts in sperm of men exposed to cigarette smoke Fertil Steril 1999 72 330 335 10.1016/S0015-0282(99)00230-7 10439006

[B5] Verhofstad N van Oostrom CT van Benthem J van Schooten FJ van Steeg H Godschalk RW DNA adduct kinetics in reproductive tissues of DNA repair proficient and deficient male mice after oral exposure to benzo(a)pyrene Environ Mol Mutagen 2009 51 2 123 9 10.1002/em.20516 19634154

[B6] Gillet LC Scharer OD Molecular mechanisms of mammalian global genome nucleotide excision repair Chem Rev 2006 106 253 276 10.1021/cr040483f 16464005

[B7] Bar-Joseph Z Siegfried Z Brandeis M Brors B Lu Y Eils R Dynlacht BD Simon I Genome-wide transcriptional analysis of the human cell cycle identifies genes differentially regulated in normal and cancer cells Proc Natl Acad Sci USA 2008 105 955 960 10.1073/pnas.0704723105 18195366 PMC2242708

[B8] Whitfield ML Sherlock G Saldanha AJ Murray JI Ball CA Alexander KE Matese JC Perou CM Hurt MM Brown PO Botstein D Identification of genes periodically expressed in the human cell cycle and their expression in tumors Mol Biol Cell 2002 13 1977 2000 10.1091/mbc.02-02-0030. 12058064 PMC117619

[B9] Cheo DL Ruven HJ Meira LB Hammer RE Burns DK Tappe NJ van Zeeland AA Mullenders LH Friedberg EC Characterization of defective nucleotide excision repair in XPC mutant mice Mutat Res 1997 374 1 9 9067411 10.1016/s0027-5107(97)00046-8

[B10] Kao SH Chao HT Wei YH Multiple deletions of mitochondrial DNA are associated with the decline of motility and fertility of human spermatozoa Mol Hum Reprod 1998 4 657 666 10.1093/molehr/4.7.657 9701788

[B11] Zhou C Li Z Diao H Yu Y Zhu W Dai Y Chen FF Yang J DNA damage evaluated by gammaH2AX foci formation by a selective group of chemical/physical stressors Mutat Res 2006 604 8 18 16423555 10.1016/j.mrgentox.2005.12.004 PMC2756993

[B12] Tripathi R Mishra DP Shaha C Male germ cell development: turning on the apoptotic pathways Journal of reproductive immunology 2009 83 31 35 10.1016/j.jri.2009.05.009 19889463

[B13] Huo J Xu S Guo K Zeng Q Lam KP Genetic deletion of faim reveals its role in modulating c-FLIP expression during CD95-mediated apoptosis of lymphocytes and hepatocytes Cell death and differentiation 2009 16 1062 1070 10.1038/cdd.2009.26 19300454

[B14] Boatright KM Salvesen GS Mechanisms of caspase activation Current opinion in cell biology 2003 15 725 731 10.1016/j.ceb.2003.10.009 14644197

[B15] Hockley SL Arlt VM Brewer D Giddings I Phillips DH Time- and concentration-dependent changes in gene expression induced by benzo(a)pyrene in two human cell lines, MCF-7 and HepG2 BMC Genomics 2006 7 260 10.1186/1471-2164-7-260 17042939 PMC1621085

[B16] Caino MC Oliva JL Jiang H Penning TM Kazanietz MG Benzo[a]pyrene-7,8-dihydrodiol promotes checkpoint activation and G2/M arrest in human bronchoalveolar carcinoma H358 cells Mol Pharmacol 2007 71 744 750 10.1124/mol.106.032078 17114299

[B17] van Oosten M Stout GJ Backendorf C Rebel H de Wind N Darroudi F van Kranen HJ de Gruijl FR Mullenders LH Mismatch repair protein Msh2 contributes to UVB-induced cell cycle arrest in epidermal and cultured mouse keratinocytes DNA Repair (Amst) 2005 4 81 89 10.1016/j.dnarep.2004.08.008 15533840

[B18] Gerlach VL Aravind L Gotway G Schultz RA Koonin EV Friedberg EC Human and mouse homologs of Escherichia coli DinB (DNA polymerase IV), members of the UmuC/DinB superfamily Proc Natl Acad Sci USA 1999 96 11922 11927 10.1073/pnas.96.21.11922 10518552 PMC18388

[B19] Ogi T Mimura J Hikida M Fujimoto H Fujii-Kuriyama Y Ohmori H Expression of human and mouse genes encoding polkappa: testis-specific developmental regulation and AhR-dependent inducible transcription Genes Cells 2001 6 943 953 10.1046/j.1365-2443.2001.00478.x 11733032

[B20] Hoogervorst EM van Oostrom CT Beems RB van Benthem J Berg J van den van Kreijl CF Vos JG de Vries A van Steeg H 2-AAF-induced tumor development in nucleotide excision repair-deficient mice is associated with a defect in global genome repair but not with transcription coupled repair DNA Repair (Amst) 2005 4 3 9 10.1016/j.dnarep.2004.08.009 15533832

[B21] de Vries A Dolle ME Broekhof JL Muller JJ Kroese ED van Kreijl CF Capel PJ Vijg J van Steeg H Induction of DNA adducts and mutations in spleen, liver and lung of XPA-deficient/lacZ transgenic mice after oral treatment with benzo[a]pyrene: correlation with tumour development Carcinogenesis 1997 18 2327 2332 10.1093/carcin/18.12.2327 9450477

[B22] Edgar R Domrachev M Lash AE Gene Expression Omnibus: NCBI gene expression and hybridization array data repository Nucleic Acids Res 2002 30 207 210 10.1093/nar/30.1.207 11752295 PMC99122

[B23] Dennis G Jr Sherman BT Hosack DA Yang J Gao W Lane HC Lempicki RA DAVID: Database for Annotation, Visualization, and Integrated Discovery Genome Biol 2003 4 P3 10.1186/gb-2003-4-5-p3 12734009 PMC193660

[B24] Huang DW Sherman BT Lempicki RA Systematic and integrative analysis of large gene lists using DAVID bioinformatics resources Nature protocols 2009 4 44 57 10.1038/nprot.2008.211 19131956

